# Supplementary material for: Prominent Josephson tunneling between twisted single copper oxide planes of Bi2Sr2-xLaxCuO6+y
Source: Nat Commun. 2023 Aug 25;14:5201. doi: 10.1038/s41467-023-40525-1 (PMC10457331; doi:10.1038/s41467-023-40525-1)
Supplement: Supplementary file 1 — Supplementary information [file 41467_2023_40525_MOESM1_ESM.pdf]

Supplementary information to:

Prominent Josephson tunneling between twisted single copper oxide planes of $Bi_2Sr_{2-x}La_xCuO_{6+y}$

Heng Wang, Yuying Zhu*, Zhonghua Bai, Zechao Wang, Shuxu Hu, Hong-Yi Xie, Xiaopeng Hu, Jian Cui, Miaoling Huang, Jianhao Chen, Ying Ding, Lin Zhao, Xinyan Li, Qinghua Zhang, Lin Gu, X.J. Zhou, Jing Zhu, Ding Zhang*, and Qi-Kun Xue*

*Corresponding author.

Email: zhuyy@baqis.ac.cn, dingzhang@mail.tsinghua.edu.cn, qkxue@mail.tsinghua.edu.cn

Contents

Supplementary Note 1. Estimation of the Josephson penetration depth

Supplementary Note 2. Determination of the twist angle by TEM

Supplementary Note 3. Isolating the transport across the twisted interface

Supplementary Note 4. Critical current in the intrinsic Josephson junctions of Bi-2201 and Bi-2212

Supplementary Note 1: Estimation of the Josephson penetration depth

The Josephson penetration depth in Bi-2201 and Bi-2212 is defined as [57]:

$$\lambda_j = \sqrt{\frac{\Phi_0 s}{2\pi\mu_0\lambda_{ab}^2 J_c}}, \quad (2)$$

where s is the thickness of the superconducting layer, μ_0 is the vacuum permeability, and λ_{ab} is the London penetration depth in the *ab*-plane. For Bi-2212, s is usually chosen as 0.3 nm [38] and λ_{ab} is about 200 nm [38, 39]. By putting $J_c = 4000$ A/cm^2 [39], one obtains $\lambda_j = 0.22$ µm. For Bi-2201, we take $s = 0.2$ nm [66] and estimate λ_{ab} of Bi-2201 to be 350 nm based on the relationship of $T_c \propto 1/\lambda_{ab}^2$ for Bi-2201 and Bi-2212 [67, 68]. We therefore evaluate λ_j to be 0.66 µm if using $J_c = 100$ A/cm^2 (see Supplementary Note 4 for the critical current density). Notably, J_c in sample S1 is 14 A/cm^2 such that $\lambda_j = 1.8$ µm, which is on the same order of magnitude as the junction width. We argue that here λ_j may be underestimated because the effective superconducting thickness often exceeds the physical layer thickness in two-dimensional superconductors [61]. For instance, in a WTe_2 monolayer, the superconducting thickness estimated from the temperature dependence of in-plane upper critical fields is 2.65 nm, whereas the physical thickness is about 0.7 nm [69].

Supplementary Note 2: Determination of the twist angle by TEM

Supplementary Figure 1 illustrates the configuration of our TEM sample holder. It can be rotated along both vertical and horizontal directions (A and B in Supplementary Fig. 1a). In order to obtain atomically resolved TEM images of high quality, the orientation of the crystal (illustrated by the red bar) should be parallel to the zone axis direction—a lattice row parallel to the intersection of two or more lattice planes. In TEM experiments, we usually tilt the sample according to the Kikuchi line pattern to align a crystal along the zone axis. The change of the crystalline orientation results in the shift of the Kikuchi pattern.

The twisted cuprate junction essentially consists of two crystals—top and bottom flakes—with different orientations. Here we focus on the situation where the twist angle is close to 45°. For a perfect alignment, the viewing direction can be simultaneously parallel to the zone axis directions of the top and bottom flakes. With a slight misalignment in the twist angle, when the viewing direction is parallel to the zone axis of the top flake, it can deviate from that of the bottom flake. In order to extract this deviation angle, we focus the electron beam on the top and bottom sections (as shown in Supplementary Fig. 2a,e), respectively. The corresponding convergent beam electron

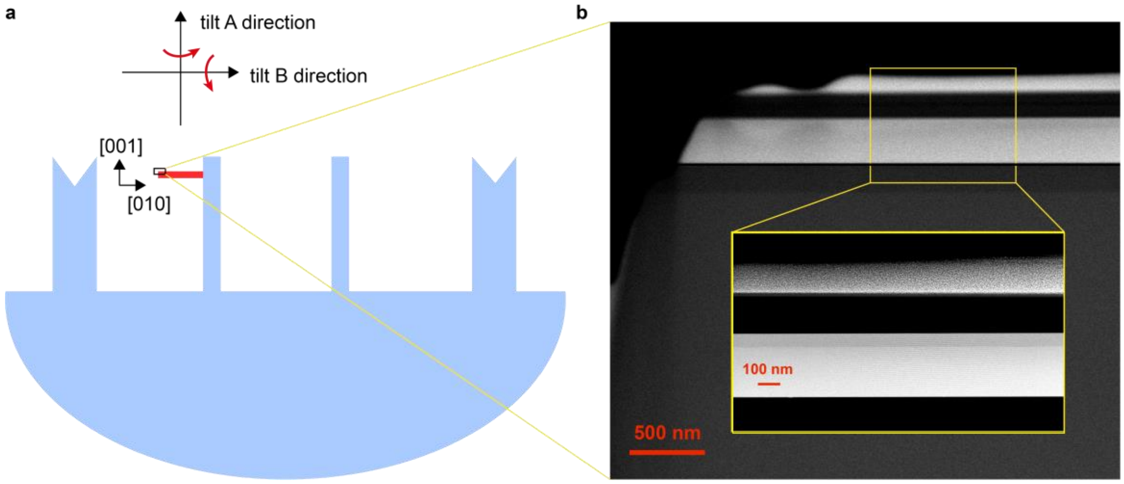

Supplementary Figure 1 TEM analysis in a wide range. a. Schematic drawing of the sample holder for cross-sectional TEM. The holder can be tilted along two directions (A and B). The red horizontal bar represents the slab cut from the BSCCO junction. **b**. Cross-sectional TEM images with large lateral sizes.

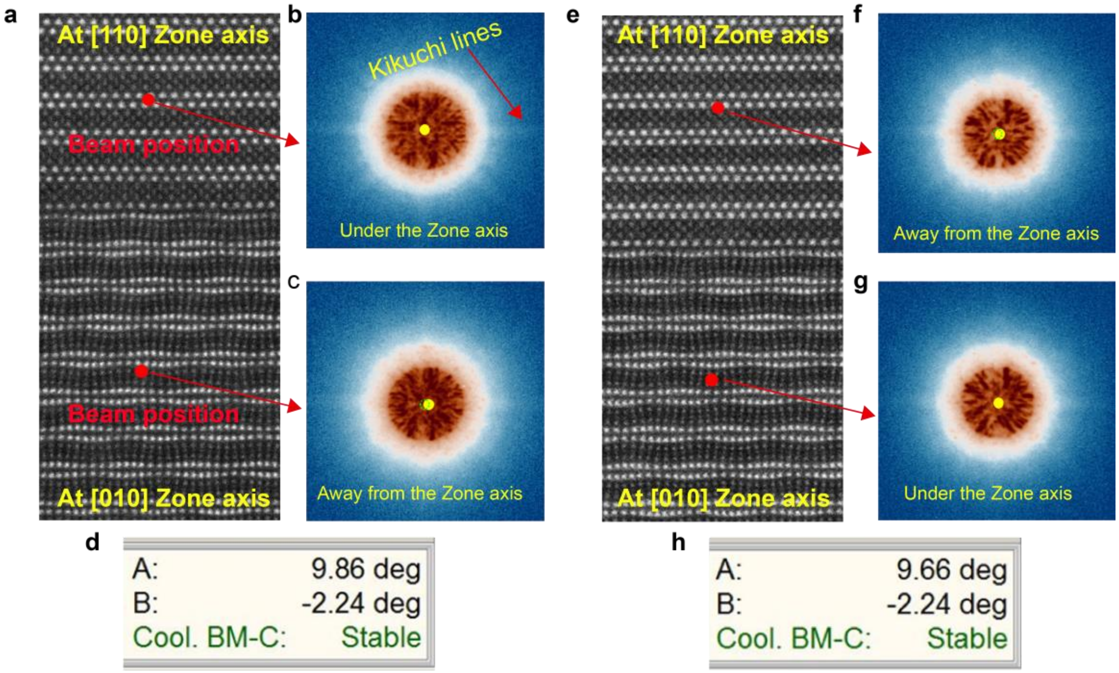

Supplementary Figure 2 Determination of the twist angle by high resolution transmission electron microscopy. a. HAADF-STEM image of the Bi-2201 junction acquired by making the upper flake under the zone axis condition according to the CBED pattern. **b** & **c**. CBED patterns of the upper and bottom flakes, respectively. The yellow solid circle shows the CoM of the CBED pattern and the green dashed circle shows the exact center of the Kikuchi line. **d**. Tilt angle of the TEM sample holder. **e**. HAADF-STEM image of the junction which is acquired by making the bottom flake under the zone axis condition according to the CBED pattern. **f** & **g**. CBED patterns of the upper and bottom flakes, respectively. **h**. Tilt angle of the TEM sample holder.

diffraction (CBED) patterns are recorded, as shown in Supplementary Fig. 2b,c and f,g. We first tilt the sample holder to align the top flake under the zone axis condition (Supplementary Fig. 2a-d). In this case, the center of mass (CoM) of the CBED pattern from the top flake is located at the exact center of the Kikuchi lines (yellow dot in Supplementary Fig. 2b). By contrast, the CoM of the CBED pattern of the bottom flake slightly deviates from the exact center (green dashed circle in Supplementary Fig. 2c) of the Kikuchi lines. Next, we tilt the sample holder such that the bottom flake is under the

zone axis condition. By doing so, the CoM of the CBED pattern of the bottom flake is located at the exact center of the Kikuchi lines (Supplementary Fig. 2g) and that of the top flake moves away from the exact center (Supplementary Fig. 2f). The angular values of the sample holder for the above two experiments are shown in Supplementary Fig. 2d and Supplementary Fig. 2h. From these values, we calculate that the deviation from perfect alignment is 9.86°-9.66°=0.20°. According to the atomically resolved TEM images, the top flake is along the [110] zone axis and the bottom flake is along the [010] zone axis. Since the nominal twist angle is 45°, we specify the twist angle as 44.8° or 45.2°.

Supplementary Note 3: Isolating the transport across the twisted interface

In this note, we argue that our bottom contacting scheme is sufficient to probe the Josephson effect just between the two twisted CuO_2 planes. Based on our fabrication, the top and bottom Bi-2201 flakes (marked as Bi-2201-t and Bi-2201-b) are contacted from the bottom by Ti/Au contacts. In order to isolate the transport exclusively from the interface, we place an additional graphite flake on the top surface of the bottom Bi-2201 flake as an electrode. Supplementary Figure 7a shows schematically the drawing of such a setup. We realize this configuration in a sample as shown in Supplementary Fig. 7b with the twist angle of 40°. In the following, we denote the voltage measured from the top flake by letter U and that from the bottom flake by V. The voltage on the top/bottom surface of the top flake is then U_t/U_b, while that on the top/bottom surface of the bottom flake is denoted as V_t/V_b. The voltage difference across the twisted interface, measured by a combination of the graphite electrode and the Ti/Au contact, is therefore $(U_b - V_t)$. No intrinsic junctions contribute to this voltage difference. In comparison, we typically measure $(U_b - V_b)$, which involves the voltage drop across the bottom flake—$(V_t - V_b)$.

Supplementary Figure 7c compares the resistances measured by using different combinations of the electrodes. Since the resistances are measured by the ac lock-in technique, we use $\widetilde{U}$ and $\tilde{V}$ to denote the corresponding voltages. however, we observe different resistance values, although the superconducting transitions are overlapping. For this sample, the vertical transport in the bottom flake gives rise to pronounced contribution, presumably due to the increased thickness of the flake.

We highlight that although the vertical transport of the bottom flake may contribute to the normal state resistance measured by $(U_b - V_b)$ at $T > T_c$, the tunneling *I*-*V* obtained in the superconducting state ($T < T_c$) solely arises from that at the interface in the small current range. This is clearly elucidated in the tunneling data in Supplementary Fig. 7d. For the $40°$-twist junction, we show that *I*-$(U_b - V_t)$ shows the Josephson

tunneling at the interface whereas $(V_t - V_b)$ stays zero in the same current range. It clearly demonstrates that the intrinsic junctions in the bottom flake stays superconducting thus does not take the voltage drop. In Supplementary Fig. 11, we show that the same sample indeed hosts a critical current of 3.5 mA for the individual flake, greatly exceeding the critical current at the twisted interface (see Supplementary Note 4).

Supplementary Note 4: Critical current in the intrinsic Josephson junctions of Bi-2201 and Bi-2212

In Supplementary Fig. 11 we study the vertical transport across a single piece of Bi-2201 flake. We use the sample shown in Supplementary Fig. 7 before. The main panel of Supplementary Fig. 11 a shows a sudden jump at a critical current of 3.5 mA, which is one order of magnitude larger than the critical current at the twisted interface. The inset shows the corresponding temperature dependence of this critical current on a single side. Based on the area of this individual flake, we estimate that the critical current density is 210 A/cm^2. This value is consistent with that reported in most of the previous works, which we summarize in Supplementary Fig. 11 b. Evidently, most of the work reported J_c values on the order of 100 A/cm^2 for Bi-2201 and 1000 A/cm^2 for Bi-2212.

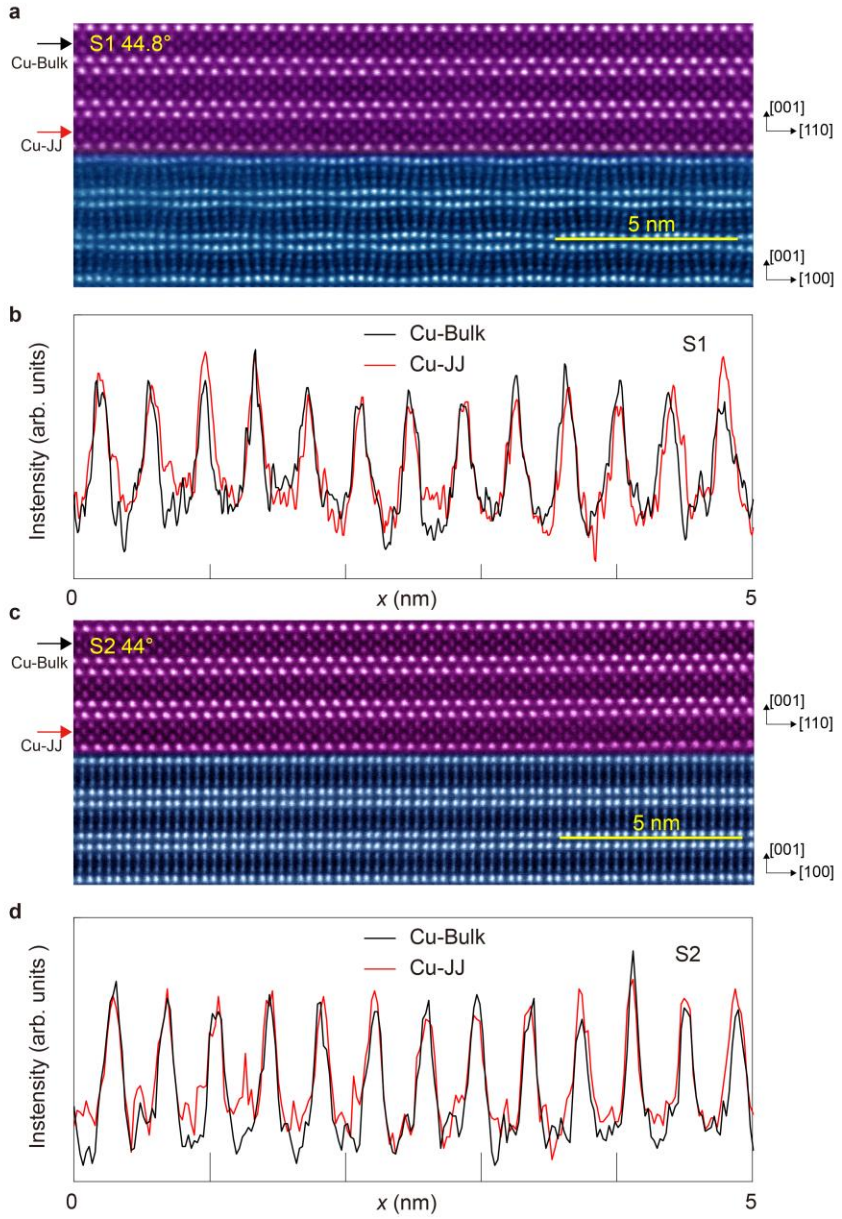

Supplementary Figure 3 Intensity analysis of the CuO_2 plane. **a** & **c**, High-resolution TEM images of S1 and S2. **b** & **d**, Intensity profiles of the CuO_2 plane in the upper flake close to the interface (indicated by the red arrows in **a** and **b**) and that in the bulk (indicated by the black arrows in **a** and **b**).

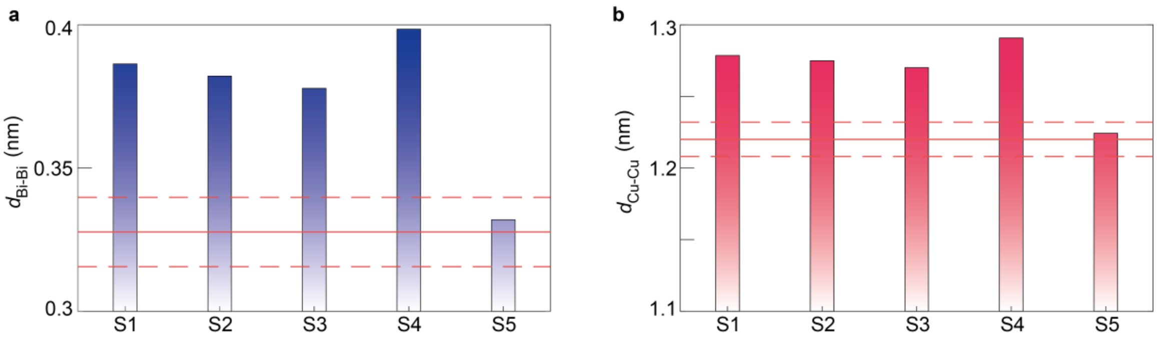

Supplementary Figure 4 Summary of interlayer distances in different samples. a and **b,** Extracted values of $d_{\text{Bi-Bi}}$ and $d_{\text{Cu-Cu}}$ in samples S1 to S5 from the TEM data. Solid lines represent the mean value of the intrinsic junction. Dashed lines indicate the error bars, as defined by the standard deviation.

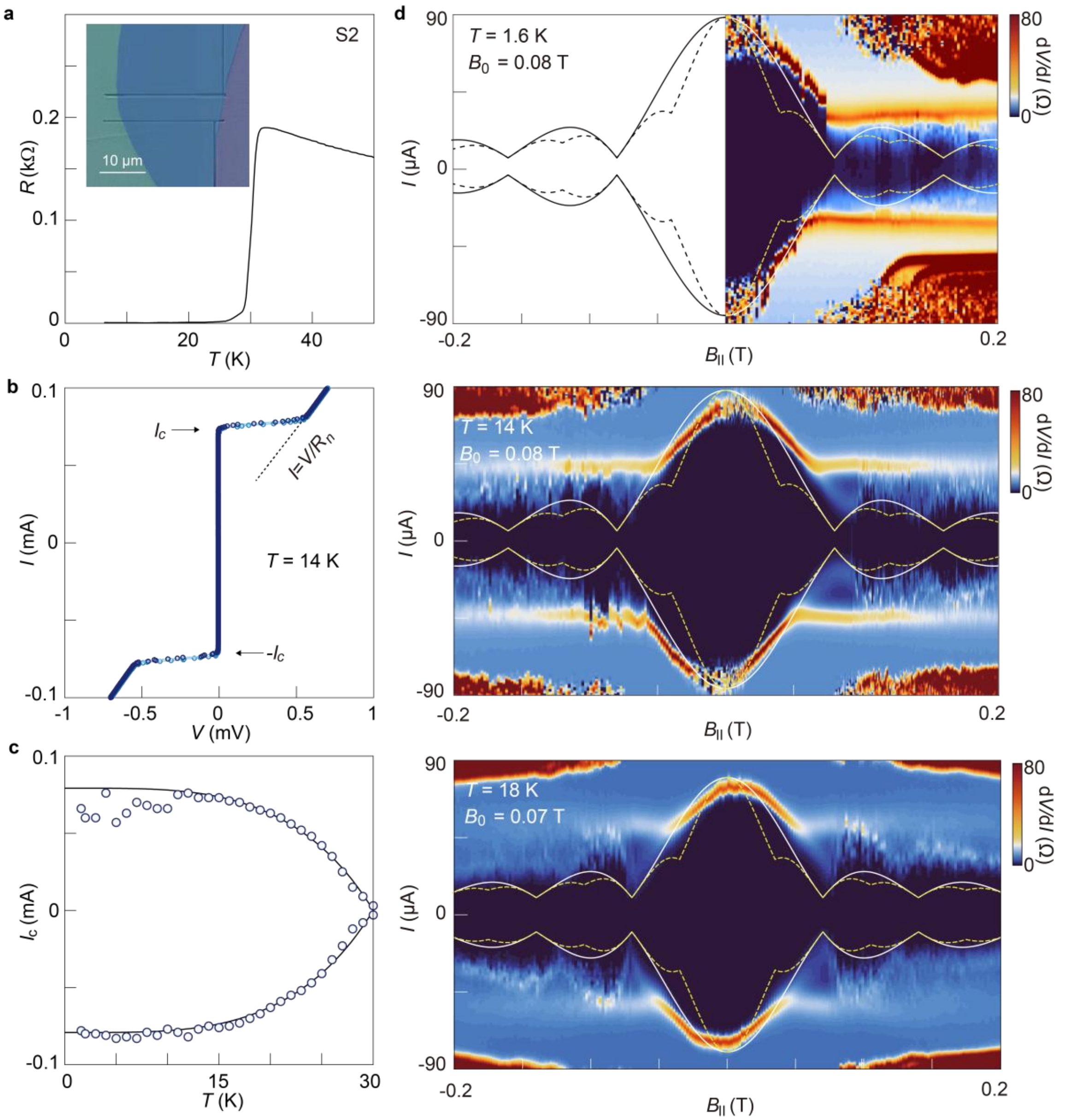

Supplementary Figure 5 Transport data of sample S2. a, Temperature dependence of the junction resistance. Inset: false-colored SEM image of S2. **b,** Tunneling characteristics at 14 K. **c,** Temperature dependence of the critical Josephson current. **d,** Color-coded dV/dI of sample S2 as a function of tunneling current and in-plane magnetic field ($B_{||}$) at three temperatures. Solid curves are from the standard formula with an offset in current. B_0 values are the periods in the magnetic field used in plotting the solid curves. Dashed curves illustrate the doubling of oscillation frequency in the Fraunhofer pattern, as expected for *d*+i*d* pairing. Here we consider 50% contribution from the co-tunneling of Cooper pairs.

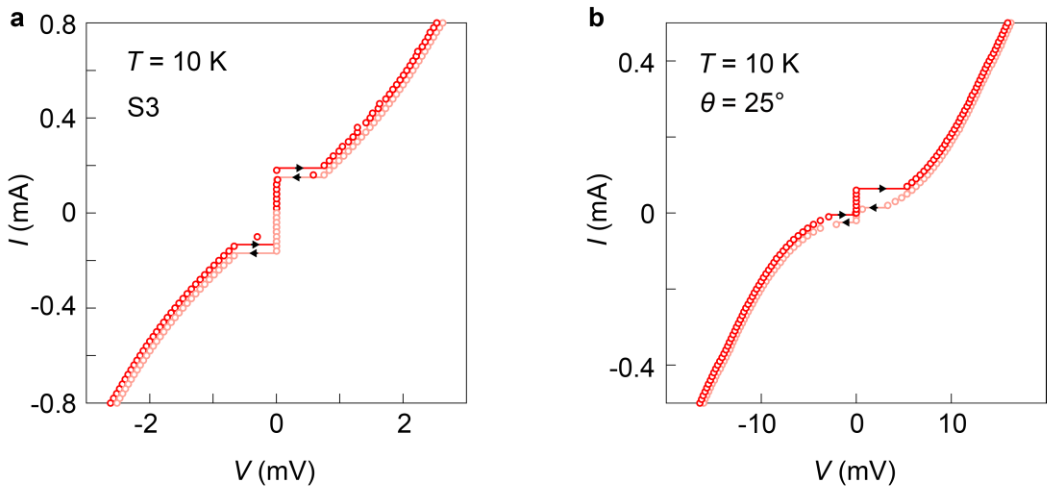

Supplementary Figure 6 Tunneling characteristics in a wide current range. a and **b,** Current-voltage curves of two twisted Bi-2201 junctions, showing a single jump in the positive or negative current direction.

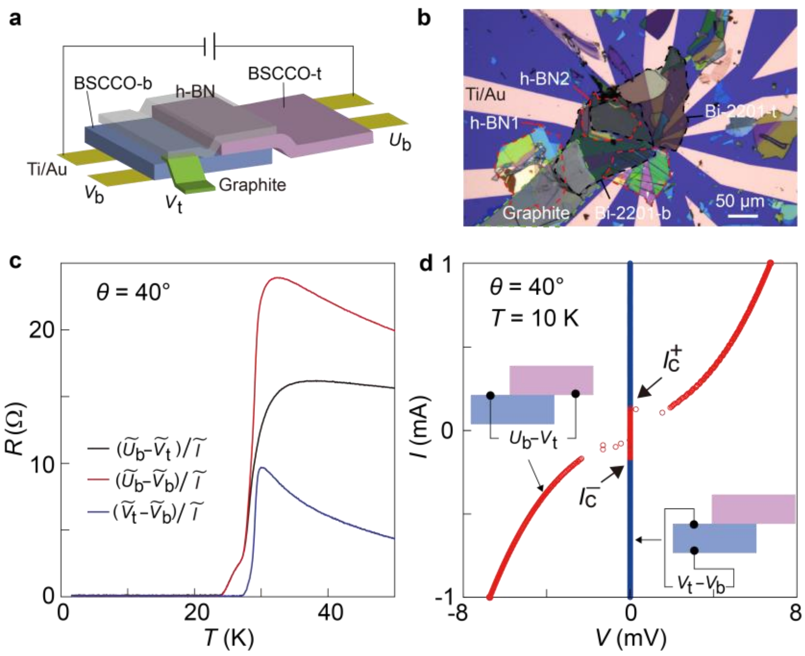

Supplementary Figure 7 Tunneling characteristics at the interface. a, Schematic drawing of device for isolating the interlayer transport from that in the bottom flake. **b,** Optical images of the two samples. The twist angle is 40°. Red curves outline the h-BN flakes on top of both Bi-2201-t and Bi-2201-b (outlined by black curves). Green curves highlight the top graphite electrode. The graphite flake is on top of h-BN-1. Another h-BN flake (h-BN-2) is used to cover the junction region. **c**, Temperature dependent resistances by using different combinations of contacts for the two samples. **d,** Interlayer tunneling characteristics of the 40°-twisted junction. Here the voltage differences are registered by using two configurations as schematically drawn in the insets.

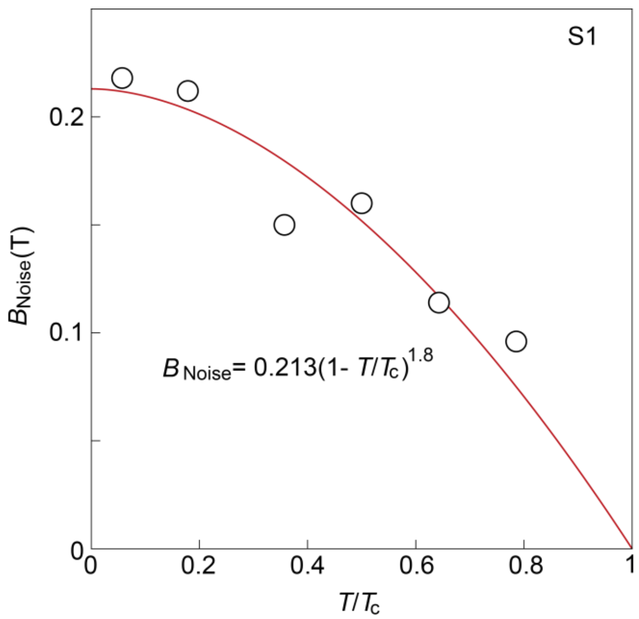

Supplementary Figure 8 Critical magnetic fields for the onset of noises in the Fraunhofer pattern of sample S1. We extract the critical magnetic field $\boldsymbol{B}_{\text{Noise}}$ at which the Fraunhofer pattern at a fixed temperature starts to become noisy (examples are shown as the red arrows in Fig. 3 of the main text). The red curve is a fitting with the fit parameters listed in the panel. We speculate that there is a slight misalignment between our junction plane and the magnetic field. The total magnetic field therefore casts a small perpendicular component. Pancake vortices enter the junction when the perpendicular magnetic field exceeds the lower critical field of Bi-2201 ($\boldsymbol{B_{c1}}$). The random jumping of these fluxes may cause instability in the Fraunhofer pattern. By using a typical $\boldsymbol{B_{c1}}$ of 0.3 mT [76], we estimate that the misalignment is 0.1°.

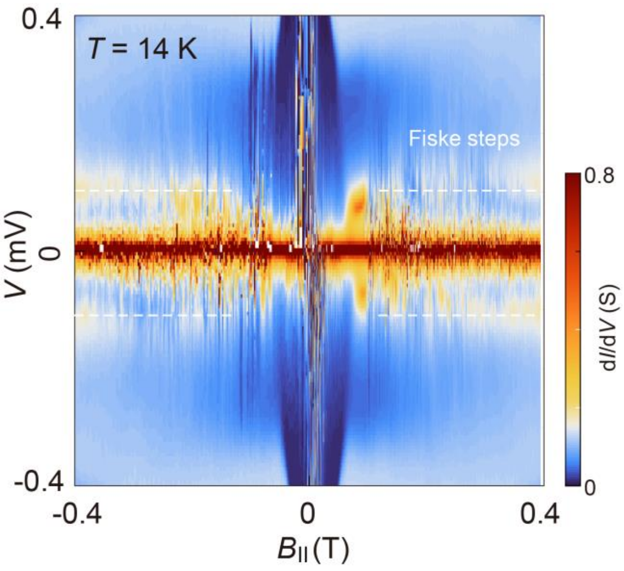

Supplementary Figure 9 Fiske steps in sample S2. Color-coded dI/dV as a function of $B_{\parallel}$ and the bias voltage across the junction at 14 K. Dashed lines mark the positions of Fiske steps.

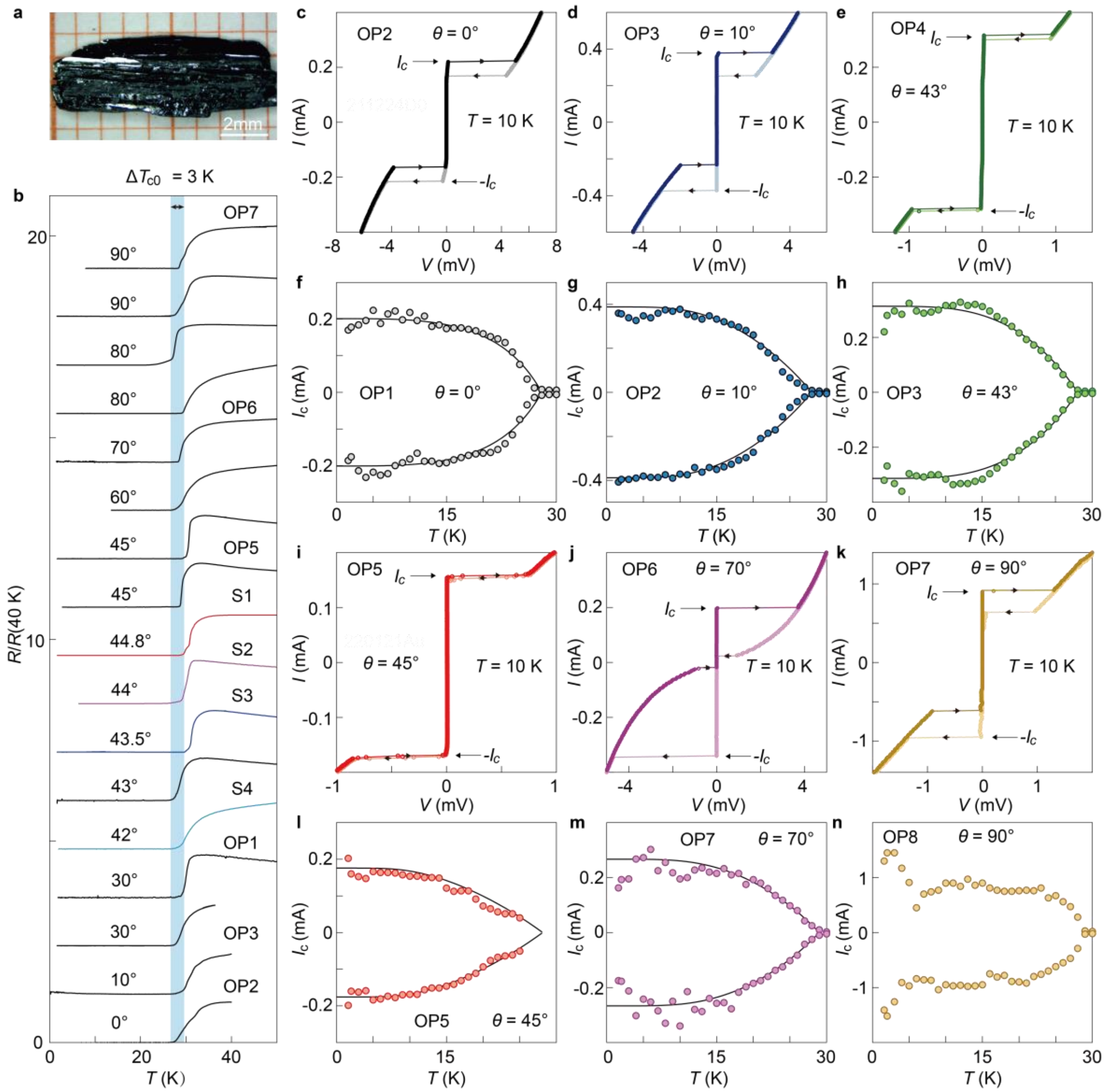

Supplementary Figure 10 Resistances and tunneling characteristics of different junctions at various twist angles. a, Optical image of the Bi-2201 single crystal used in this work. **b**, Temperature dependent junction resistances samples exfoliated from the same crystal. The resistance of each sample is normalized by its value at 40 K. Curves are vertically offset for clarity. The shaded blue stripe indicates the variation in T_c. **c**-**e**, **i-k,** Current-voltage tunneling characteristics at *T* = 10 K. Dark and light colors represent data from two opposite sweeping directions, as indicated by the arrows. **f**-**h, l-n,** Temperature dependence of the Josephson critical current. Black curves are guide to the eye by using a modified Ambegaokar-Baratoff formula [6].

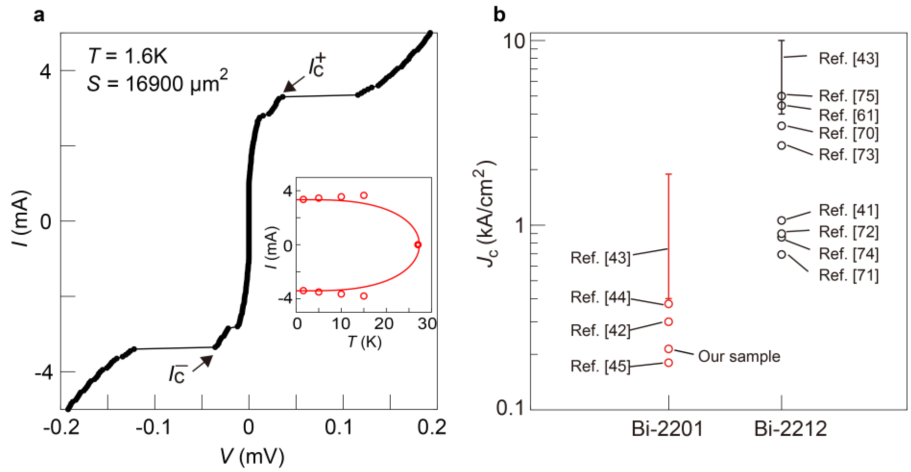

Supplementary Figure 11 Critical current in the intrinsic Josephson junctions. a, *I-V* characteristic across the bottom flake of a Bi-2201 junction (shown in Supplementary Fig. 7). Inset shows the temperature dependence of the critical current (circles). Solid curves are guide to the eye by using a modified Ambegaokar-Baratoff formula [6]. **b,** Summary of the reported critical current density values in Bi-2201 [42-45] and Bi-2212 [41, 43, 61, 70-75] intrinsic junctions. We note that one report [43] gives exceptionally high critical current density for both Bi-2201 (2 kA/cm^2) and Bi-2212 (10 kA/cm^2). Most of the experiments report only a few kA/cm^2 for Bi-2212 and hundreds of A/cm^2 for Bi-2201.

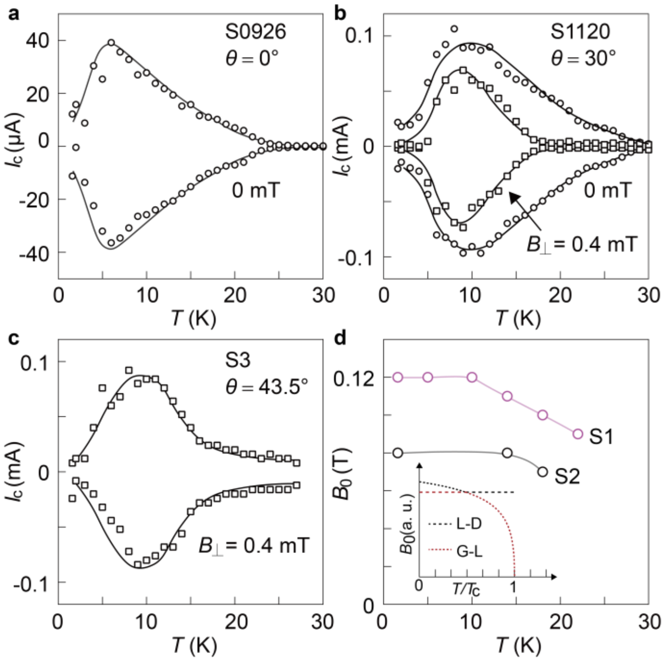

Supplementary Figure 12 Temperature dependence of the critical current and Fraunhofer period. a-c, $I_c(T)$ of three samples with different twist angles. The magnetic field is applied perpendicular to the sample in **b** and **c**. Solid curves are guide to the eye. **d**, Temperature dependence of the extracted B_0 from S1 and S2. Inset shows the theoretical trend. Dashed and dotted curves represent the theoretically expected B_0 from the Lawrence-Doniach (L-D) and Ginzburg-Landau (G-L) models, respectively. Red parts indicate the crossover from the L-D regime to the G-L regime.

[66] S. O. Katterwe, Th. Jacobs, A. Maljuk, and V. M. Krasnov, Low anisotropy of the upper critical field in a strongly anisotropic layered cuprate $Bi_{2.15}Sr_{1.9}CuO_{6+\delta}$: Evidence for a paramagnetically limited superconductivity. *Phys. Rev. B* **89**, 214516 (2014).

[67] R. Khasanov, T. Kondo, S. Strässle, D. O. G. Heron, A. Kaminski, H. Keller, S. L. Lee, and T. Takeuchi, Evidence for a competition between the superconducting state and the pseudogap state of $(BiPb)_2(SrLa)_2CuO_{6+\delta}$ from muon spin rotation experiments. *Phys. Rev. Lett.* **101**, 227002 (2008).

[68] Y. J. Uemura, G. M. Luke, B. J. Sternlieb, J. H. Brewer, J. F. Carolan, W. N. Hardy, R. Kadono, J. R. Kempton, R. F. Kiefl, S. R. Kreitzman, P. Mulhern, T. M. Riseman, D. L. Williams, B. X. Yang, S. Uchida, H. Takagi, J. Gopalakrishnan, A. W. Sleight, M. A. Subramanian, C. L. Chien, M. Z. Cieplak, G. Xiao, V. Y. Lee, B. W. Statt, C. E. Stronach, W. J. Kossler, and X. H. Yu, Universal correlations between T_c and n_s/m* (Carrier density over effective mass) in High-T_c cuprate cuperconductors. *Phys. Rev. Lett.* **62**, 2317 (1989).

[69] V. Fatemi, S. Wu, Y. Cao, L. Bretheau, Q. D. Gibson, K. Watanabe, T. Taniguchi, R. J. Cava, P. J.-Herrero, Electrically tunable low-density superconductivity in a monolayer topological insulator. *Science* **362**, 922 (2018).

[70] K. Inomata, S. Sato, K. Nakajima, A. Tanaka, Y. Takano, H. B. Wang, M. Nagao, H. Hatano, and S. Kawabata, Macroscopic Quantum Tunneling in a *d*-Wave High-T_c $Bi_2Sr_2CaCu_2O_{8+\delta}$ Superconductor. *Phys. Rev. Lett.* **95**, 107005 (2005).

[71] X. B. Zhu, Y. F. Wei, S. P. Zhao, G. H. Chen, H. F. Yang, A. Z. Jin, and C. Z. Gu, Intrinsic tunneling spectroscopy of $Bi_2Sr_2CaCu_2O_{8+\delta}$: The junction-size dependence of self-heating. *Phys. Rev. B* **73**, 224501 (2006).

[72] K. Kadowaki, I. Kakeya, T. Yamamoto, T. Yamazaki, M. Kohri, Y. Kubo, Dynamical properties of Josephson vortices in mesoscopic intrinsic Josephson junctions in single crystalline $Bi_2Sr_2CaCu_2O_{8+\delta}$. *Physica C*, **437**, 111 (2006).

[73] X. Y. Jin, J. Lisenfeld, Y. Koval, A. Lukashenko, A. V. Ustinov, and P. Müller, Enhanced Macroscopic Quantum Tunneling in $Bi_2Sr_2CaCu_2O_{8+\delta}$ Intrinsic Josephson Junction Stacks. *Phys. Rev. Lett.* **96**, 177003 (2006).

[74] M. Suzuki, R. Takemura, K. Hamada, M. Ohmaki, and T. Watanabe, Short-Pulse Intrinsic Tunneling Spectroscopy in $Bi_2Sr_2CaCu_2O_{8+\delta}$ under Suppressed Self Heating. *Jpn. J. Appl. Phys.* **51**, 010112 (2012).

[75] S. Umegai, A. Yamaguchi, Y. Kakizaki, D. Kakehi, and H. Kitano, Fabrications of Small and High-quality Intrinsic Josephson Junctions by Combinatorial Method of Ar-ion and Focused Ga-ion Etchings. *J. Phys.: Conf. Ser.* **1054**, 012030 (2017).

[76] S. Tochihara, H. Yasuoka, H. MazakiLower, M. Osada, and M. Kakihana. Critical fields of $Bi_2Sr_2CaCu_2O_{8+d}$ and $YBa_2Ca_3O_{7-d}$ single crystals. *J. of Appl. Phys.* **85**, 8299 (1999).
